# Spontaneous catheter tip migration in Totally Implantable Venous Access Port (TIVAP) to right atrium

**DOI:** 10.1016/j.ijscr.2024.110122

**Published:** 2024-08-08

**Authors:** Ni Gusti A.A. Manik Yuniawaty Wetan, Hendry Irawan, Putu Anda Tusta Adiputra

**Affiliations:** Surgical Oncology Division, Department of Surgery, Udayana University, Prof IGNG. Ngoerah General Hospital, Denpasar, Bali, Indonesia

**Keywords:** TIVAP, Complication, False route, Spontaneous migration

## Abstract

**Introduction and importance:**

The use of a Totally Implantable Venous Access Port (TIVAP) has been a popular access option in chemotherapy for cancer patients, but complications, both long-term and short-term, may arise in the fixation process. This paper discusses the importance of detection and management of complications that arise as a result of TIVAP insertion.

**Case presentation:**

A 51-year-old female patient came to the hospital to undergo a TIVAP implantation for her chemotherapy through the right subclavian vein; however, a false route occurred to the right internal jugular vein instead of the right atrium. No direct revision was conducted for this case. Follow-up was conducted for 3 months. The first month post-chemotherapy, the access flow remained smooth. No complications were found.

**Clinical discussion:**

The right subclavian vein is preferred for its low complication and high success rates. Typically, a C-Arm is used to guide TIVAP insertion, allowing immediate corrections to prevent complications. In this patient, the C-Arm was unavailable due to logistical constraints. The TIVAP catheter tip was evaluated and sustained. High-velocity flow at the catheter tip can cause a jet effect, potentially shifting the catheter tip cranially or to the proper position in the right atrium.

**Conclusion:**

We conclude that TIVAP attachment does not have to be redone when a false route happens, and routine observation on possible complications as well as gradual drug administration should be done instead.

## Highlights

• Totally Implantable Venous Access Port (TIVAP) catheter malposition
• Management of TIVAP Malposition
• Preferred Vein for TIVAP

## 1 Introduction

The use of Totally Implantable Venous Access Port (TIVAP) is growing in popularity and has made advancements in treatment plans as well as the quality of life of cancer patients undergoing chemotherapy. TIVAP creates better comfort for patients needing long term vein access, especially those who undergo cytotoxic treatments such as chemotherapy by preventing complications such as pain, phlebitis, repetitive needle injections, and cosmetic concerns [[1], [2], [3]]. TIVAP is not only used in chemotherapy, but also in taking blood samples, administering parenteral nutrition, transfusion, administration of intravenous medications, and hemodyalisis [2,3]. The principle of using TIVAP for hemodialysis is the same, but the catheter size is different. For dialysis, a larger catheter is used. In the study by Yeum et al., which involved 132 uremic patients and a total of 150 attempts at internal jugular cannulation, the overall success rate was 90.9 %, with an average of 2.3 ± 2.1 puncture attempts. Of these attempts, 124 (82.7 %) were performed on the right side and 26 (17.3 %) on the left. The findings indicate that internal jugular vein catheterization is relatively safe and effective for providing temporary vascular access for hemodialysis [4].

**Fig. 1:**
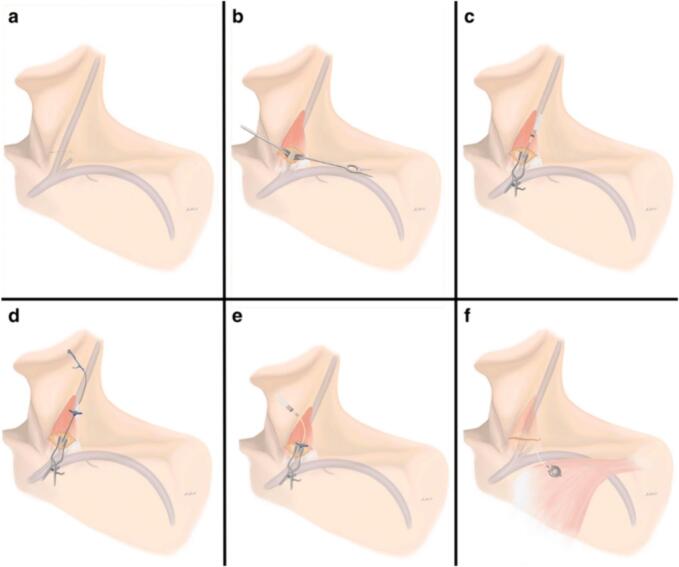
TIVAP insertion.

There is still a current debate in the clinical implementation of TIVAP regarding the insertion area (internal jugular vein and subclavian vein) and the insertion technique (opened, percutaneous, or with USG guidance). Fig. 1 Demonstrate the procedure of inserting TIVAP. The subclavian vein is the vein most commonly used in TIVAP insertion. More than 5 million patients in the US undergo TIVAP insertion with 6.2–10.7 % experiencing serious complications [5]. Two types of TIVAP complications can happen, being the early complications, including pneumothorax, haemothorax, air embolism, arterial puncture, cardiac arrhythmia, pericardial tamponade, catheter malposition, and brachial plexus injury, and long-term complications, including infection, thrombosis, catheter dysfunction, migration, erosion and vena cava superior perforation, and port inversion [3,[5], [6], [7], [8]] (Table 1).

**Table 1:** Early and late complication of TIVAP insertion.

Early complication		Late complication	
Catheter insertion	Reservoir implantation	Catheter related	Reservoir related
Pneumothorax	Wound dehiscence	Catheter occlusion	Reservoir fracture
Hemothorax	Hematoma	Catheter misplacement	Reservoir rotation
Arterial puncture hematoma	Wound INFECTION	U-DVT	Drug extravasation
		Pinch-off syndrome	

Complications are also classified based on the insertion process. Complications related to port insertion include pneumothorax, hemothorax, arterial puncture, and air embolism. Complications from reservoir insertion and use include wound dehiscence and local extravasation. Complications from indwelling catheters include port malfunction, catheter migration, and embolization [7].

**Fig. 2:**
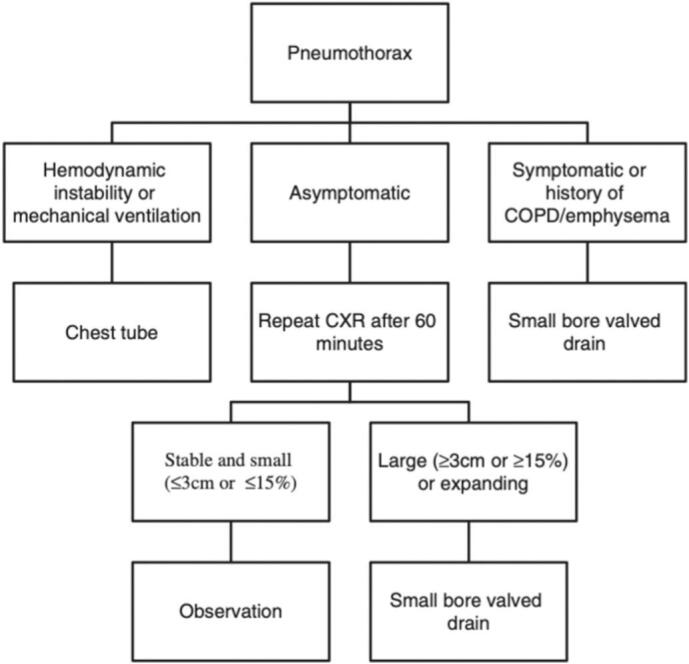
Pneumothorax management.

**Fig. 3:**
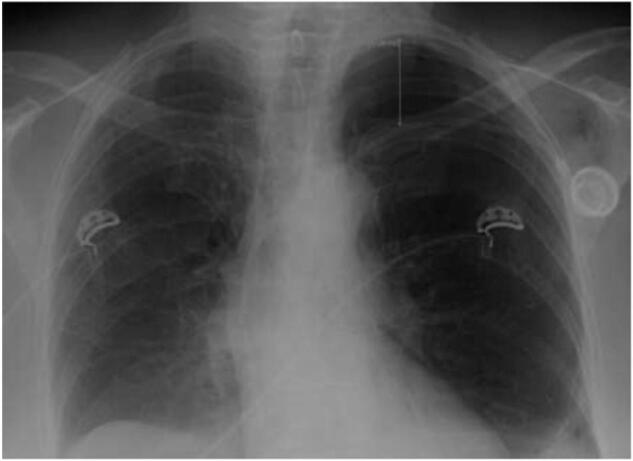
Chest X-ray of a patient with pneumothorax.

Complications related to port insertion include pneumothorax, hemothorax, arterial puncture, and air embolism. Pneumothorax happens when the lung pleura is accidentally punctured during catheter insertion, allowing air to enter the pleural cavity. Inserting a catheter into the subclavian vein is a common cause of pneumothorax due to the nearby anatomy. The management of pneumothorax is determined by the patient symptoms like in Fig. 2. The chest x-ray of pneumothorax is displayed in Fig. 3. After any central venous access, a chest X-ray (CXR) is needed to check for complications. Unstable patients should receive a large bore chest tube. Asymptomatic patients with small pneumothorax (<3 cm) can go home if a follow-up CXR shows no increase in size. However, patients with emphysema or those needing mechanical ventilation should be treated more aggressively, regardless of size. Symptomatic patients or those with larger pneumothoraces should get a small bore chest tube [7].

Hemothorax is a rare complication that can happen if arteries or veins are accidentally punctured during central venous catheter placement. Each side of the chest can hold 2 to 3 L of blood, which can cause low blood pressure if not managed. The right internal jugular vein is easier for catheter placement, but this increases the risk of hemothorax due to nearby structures. If sudden shortness of breath and low blood pressure occur during or after the procedure, hemothorax should be suspected. Ultrasound can detect as little as 50 mL of blood, while a chest X-ray might miss >300 mL. Treatment involves placing a chest tube and resuscitating the patient. If >1500 mL is drained or if bleeding is over 200 to 300 mL in the first few hours, surgery is needed. Another option is arterial embolization by interventional radiology [7].

Air embolism is a very rare but serious complication of port insertion. When breathing normally, the pressure in the chest drops slightly, creating suction that helps blood flow. Symptoms depend on how much air gets in and can include low oxygen levels, sudden shortness of breath, or even total circulatory collapse and death. An EKG may show signs of strain on the right side of the heart. Transesophageal echocardiography is the best way to diagnose this issue. Giving supplemental oxygen is crucial to prevent lung problems and help absorb the trapped air. Patients should be positioned lying down with the left side tilted down to keep the air bubble away from the right ventricle. If the catheter is in the right heart, aspiration might be attempted [7].

The internal carotid artery is punctured in about 3 % of internal jugular vein catheterizations, while subclavian artery injuries happen in about 0.5 % of subclavian vein procedures. To avoid serious complications, it's important to focus on anatomical landmarks, stay alert for arterial punctures, and apply pressure quickly. Using ultrasound for internal jugular vein catheterization is especially recommended for obese patients [7].

Complications from reservoir insertion and use include wound dehiscence and local extravasation. Wound dehiscence can occur after port placement due to technical errors or poor healing from cancer, malnutrition, or chemotherapy. Extravasation of chemotherapy drugs happens in about 0.1 % to 6 % of patients, usually when the infusate leaks into surrounding tissues due to catheter issues or reservoir failure. Symptoms depend on the type of leaked medication; irritant drugs cause pain and inflammation, while vesicants can lead to severe tissue damage. Early signs include swelling, redness, pain, burning, slow infusion rates, and trouble drawing blood from the port. Management involves quickly identifying the issue and stopping any further infusion. The needle should only be removed after thoroughly aspirating the infusate [7].

**Fig. 4:**
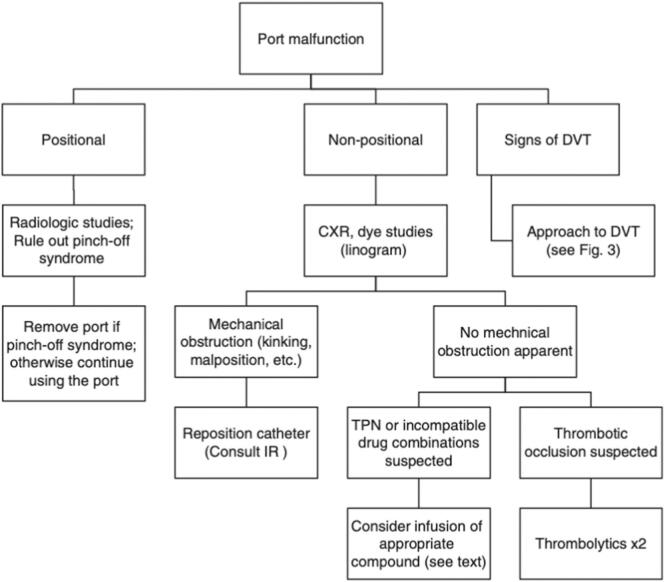
Management of port malfunction.

**Fig. 5:**
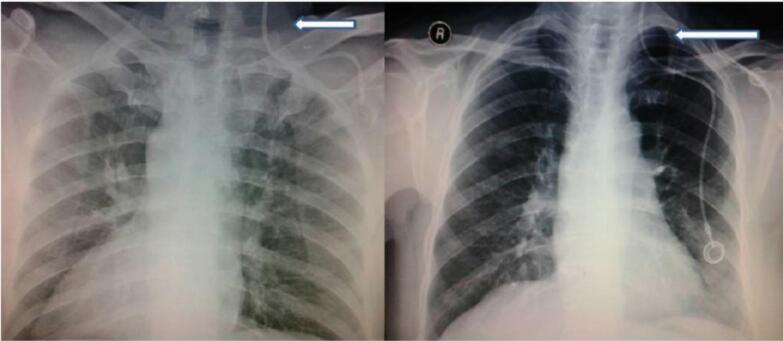
Catheter malposition to internal jugular vein during insertion.

Complications from indwelling catheters include port malfunction, catheter migration, and embolization. Port malfunction can mean difficulty using the device for blood aspiration or drug injection, with rates varying from 0 % to 47 %. Other issues include blood clots in the catheter or veins, such as deep vein thrombosis (DVT). To check for mechanical obstruction, imaging like a chest X-ray (CXR) is needed, especially if the blockage is positional. Using contrast agents can help improve visibility. Some obstructions can be treated with radiology instead of removing the port. Pinch-off syndrome is a rare issue that occurs when the catheter is pressed between the first rib and the clavicle, causing blockage that can be resolved by raising the arm on the same side. Thus, a CXR should be done with the arms at the sides, not raised. Treatment for drug precipitation in the catheter depends on the specific drug involved. Catheters can get damaged from pinch-off syndrome or external forces, like sudden stops from a seatbelt, rubbing against tight clothes, or forceful flushing with small syringes (<10 mL). Diagnosis is easy with chest X-rays. Ports should be placed deep enough, especially if a patient is likely to lose weight. Catheters shouldn't sit over bony areas and should be as straight as possible. If a catheter fragment is found, the port should be removed, and the fragments retrieved by interventional radiology, usually through the femoral vein, to prevent further issues [7] (Fig. 4, Fig. 5).

With the importance of detection and management of complications that arise as a result of TIVAP insertion. The study aims to highlight the clinical implications, possible causes, diagnosis, management strategies, and potential complications associated with such migrations to inform and improve clinical practice and patient outcomes.

## 2 Case

A 51-year-old female came to the hospital to undergo TIVAP implantation. She was previously diagnosed with colon adenocarcinoma and is scheduled to undergo chemotherapy with Curacil 500 mg and Irenotecan 40 mg regimen. History of diabetes, hypertension, lung disease, and heart disease is denied. Physical examination is within normal limits. Laboratory tests show that kidney and liver functions are normal.

**Fig. 6:**
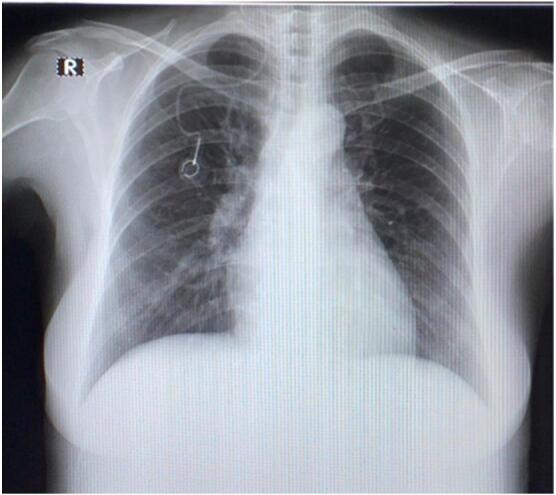
Thoracic X-ray post-TIVAP insertion.

**Fig. 7:**
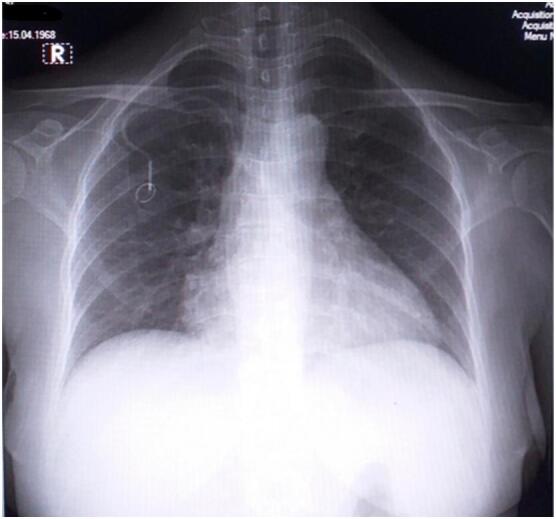
Thoracic X-ray 1-month post-TIVAP insertion.

**Fig. 8:**
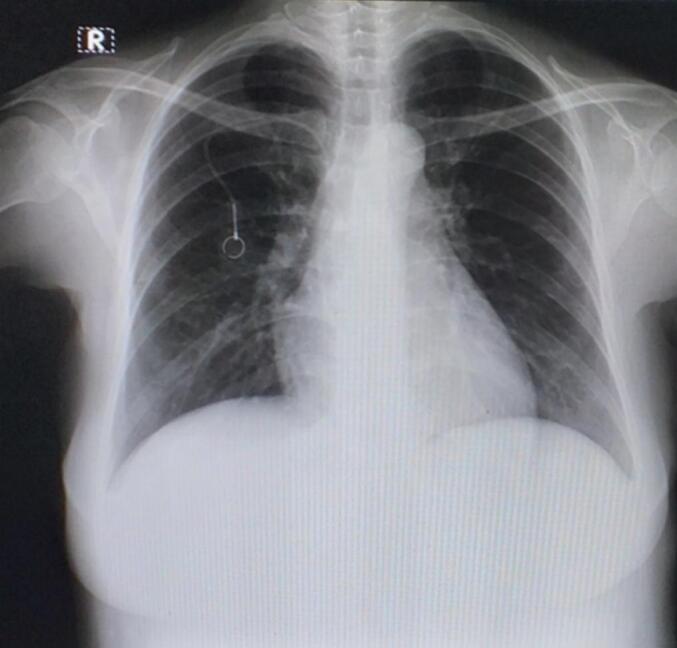
Thoracic X-ray 2-months post-TIVAP insertion.

The right subclavian vein is chosen as the vein access for the TIVAP insertion. One month later, a control thoracic X-ray after TIVAP insertion has found a malposition with the catheter tip going towards the internal jugular vein instead of the right atrium, as seen in Fig. 6. Even though there was a malposition, no replacements or corrections were directly made, and routine observation and drug administration were continued post-TIVAP. Each time chemotherapy is administered, it is first ensured that the access is functioning well and the flow is smooth, followed by an X-ray taken every month for three consecutive months. No complications as a result of chemotherapy administration were found post-evaluation. A control X-ray was taken a month after TIVAP positioning, and the catheter tip was found to be in its' appropriate location, which is the right atrium, as seen on Fig. 7. 2 months after chemoport installation, the patient conducted a bone survey, and the thoracic X-Ray picture shows a properly placed TIVAP catheter tip in its normal position, as seen in Fig. 8.

## 3 Discussion

TIVAP insertion was initiated in this patient through her right subclavian vein. There is still an ongoing debate on the most appropriate vein for TIVAP insertion, and the right subclavian vein is currently the first choice with low complications and high success rates [2].

This patient experienced a TIVAP catheter misposition to the right internal jugular vein instead of the right atrium. Ideally, TIVAP insertion is guided with the C Arm tool so that when malposition or a false route happens, an immediate correction can be made to prevent unwanted complications. However, C-Arm was not used in this patient due to logistical limitations. In this patient, the placement was performed using ultrasound guidance. Generally, the complications of TIVAP insertion can be divided into early and late complications. A study done by Yusup et al. found that, from 2080 patients who underwent TIVAP insertions, 37 patients experienced early complications in the form of catheter tip misposition to the contralateral, or ipsilateral jugular vein, and repositioning was made using fluoroscopy in the angiography unit [2]. Research conducted by Wu et al. illustrated that 19.32 % (298/1542) patients experience complication in TIVAP implantation. Early complications include catheter misposition, improper reservoir containment, cutaneous infections, and vessel leaks. Etiology of this misposition includes intra-catheter thrombosis or precipitation, fibrin sheath formation or venous thrombosis around the catheter, catheter kinking, pinch-off syndrome and catheter migration. Catheter misposition is found to happen in 2.59 % (5/234) TIVAP cases inserted through the subclavian vein [8]. Selami et al. has found that 1.23 % (4/423) patients experience malposition of the catheter tip, of where one was resolved in the angiography lab, and the rest were directly corrected with the help of fluoroscopy [9].

The external (30 %) and internal (5.7 %) jugular veins are common sites for TIVAP mispositions [10]. In this patient, a thoracic X-ray was conducted to evaluate post-TIVAP conditions. Mispositions of migrations may happen asymptomatically, hence, monitoring should be conducted periodically in the form of thoracic X-ray as a method of early detection [10]. Misposition can happen immediately after insertion or later on after a period of time due to spontaneous catheter tip movement. Risk factors of catheter tip misposition include anatomical abnormalities of the blood vessels, which are typically hereditary, or other factors unrelated to anatomy, such as insertion sites, insertion techniques, and the patient's position during insertion. The management of catheter tip malposition depends on the location of the catheter, the indication of insertion, and patients conditions [4].

This case is similar to a study by Wong et al., who reported a rare instance of TIVAP dysfunction due to a catheter migrating into the pleural space. This study present a case of a 70-year-old man with TIVAP dysfunction caused by a rare extra-vascular migration of the catheter into the pleural space, diagnosed via computed tomography. After discussing with the patient and his primary care oncologist, the decision was made to observe without further use of the TIVAP and to switch to oral chemotherapy. Leaving the TIVAP without further use did not cause any adverse effects; therefore, this option was also provided [9].

Even though a mispositioning of the internal jugular vein has been observed, no immediate repositioning was done in this patient. The TIVAP catheter tip was sustained while the evaluation was made. No issues were recognized in the evaluation process; hence, we continued on the gradual delivery of chemotherapy drugs through TIVAP. Observation post-chemotherapy also resulted in no signs of complications; hence, the catheter tip was not moved. A month after the TIVAP insertion, a thoracic X-ray was conducted, and oddly, the catheter tip spontaneously progressed towards the right atrium [6]. No complications were seen post-TIVAP insertion or chemotherapy as the catheter progressed. The presence of high-velocity flow on the tip of the catheter may result in a jet effect, resulting in a shift of the catheter tip position cranially or to its' appropriate position, which is the right atrium. Through this case, it can be concluded that the placement of a TIVAP catheter is safer and minimally invasive with ultrasound guidance. In this case, the Seldinger technique was used (with ultrasound guidance). Although we did not use C-Arm during the operation due to the lack of fluoroscopy or portable C-Arm facilities, we performed C-Arm imaging immediately after the procedure in the radiology room before using the access for chemotherapy. If mispositioning occurs, it may be considered not to directly manage a false route for the TIVAP, while also ensuring thorough observation and monitoring for possible complications. In developed countries, minimally invasive techniques (such as percutaneous or Seldinger techniques) are widely used, especially in areas with ultrasound facilities. However, in regions without ultrasound guidance, it is recommended to use conventional methods, specifically open techniques instead of blind techniques.

The strengths of this study include a detailed case presentation, comprehensive integration of existing literature, and high clinical relevance, particularly in managing TIVAP insertions for chemotherapy. It offers valuable insights and practical guidance on handling catheter misposition without immediate correction, which can inform clinical decision-making. However, the study's limitations are it is based on a single patient case, limiting the generalizability of its findings. The lack of C-Arm utilization due to logistical constraints restricts comparisons with standard practices where C-Arm is used, potentially affecting the conclusions about the safety and efficacy of not repositioning the catheter. Additionally, the short-term follow-up period (one month) may not capture long-term complications or outcomes, limiting the understanding of long-term safety and efficacy. This work has been reported in line with the scare criteria [10].

## 4 Conclusions

The use of a Totally Implantable Venous Access Port (TIVAP) is a crucial modality as access for chemotherapy. Several complications may arise during its' implementation, one of them being a false route. This can be minimized with ultrasound guidance during the placement Physicians and surgeons need to be aware of the risk when inserting a TIVAP and know how to prevent it by checking the catheter tip before use or replacement. This case shows that no immediate resolution is necessary, but period observation regarding possible complications and gradual chemotherapy administration should be conducted.

## Informed consent

Written informed consent for the case report publication was obtained from the patient.

## Ethical approval

The article has been reviewed by Hospital Legal and Ethics Committee.

## Funding

This research did not receive any specific grant from funding agencies in the public, commercial, or not-for-profit sectors.

## Author contribution

Ni Gusti AA. Manik Yuniawaty Wetan

- Contributing to the conceptualization and design, conducting data collection, analyzing and interpreting the data, writing the paper.

Hendry Irawan

- Contributing to the conducting data collection, analyzing and interpreting the data

Putu Anda Tusta Adiputra

- Contributing to the conceptualization and design, analyzing and interpreting the data.

## Guarantor

Ni Gusti AA. Manik Yuniawaty Wetan.

## Conflict of interest statement

No conflict of interests was recognized.
